# Methionine Synthase A2756G Polymorphism and Risk of Colorectal Adenoma and Cancer: Evidence Based on 27 Studies

**DOI:** 10.1371/journal.pone.0060508

**Published:** 2013-04-09

**Authors:** Weixing Ding, Dong-lei Zhou, Xun Jiang, Lie-sheng Lu

**Affiliations:** Department of Gastroenterology, The Tenth People’s Hospital of Tongji University, Shanghai, People’s Republic of China; Centro di Riferimento Oncologico, IRCCS National Cancer Institute, Italy

## Abstract

Methionine synthase (MTR), which plays a central role in maintaining adequate intracellular folate, methionine and normal homocysteine concentrations, was thought to be involved in the development of colorectal cancer (CRC) and colorectal adenoma (CRA) by affecting DNA methylation. However, studies on the association between MTR A2756G polymorphism and CRC/CRA remain conflicting. We conducted a meta-analysis of 27 studies, including 13465 cases and 20430 controls for CRC, and 4844 cases and 11743 controls for CRA. Potential sources of heterogeneity and publication bias were also systematically explored. Overall, the summary odds ratio of G variant for CRC was 1.03 (95% CI: 0.96–1.09) and 1.05 (95% CI: 0.99–1.12) for CRA. No significant results were observed in heterozygous and homozygous when compared with wild genotype for these polymorphisms. In the stratified analyses according to ethnicity, source of controls, sample size, sex, and tumor site, no evidence of any gene-disease association was obtained. Results from the meta-analysis of four studies on MTR stratified according to smoking and alcohol drinking status showed an increased CRC risk in heavy smokers (OR = 2.06, 95% CI: 1.32–3.20) and heavy drinkers (OR = 2.00, 95% CI: 1.28–3.09) for G allele carriers. This meta-analysis suggests that the MTR A2756G polymorphism is not associated with CRC/CRA susceptibility and that gene-environment interaction may exist.

## Introduction

Colorectal cancer (CRC) is the third most common malignancy and the fourth most frequent cause of cancer deaths worldwide. More than one million new cases of CRC are diagnosed annually, and nearly 530,000 individuals die from CRC every year [1]. The etiology of CRC is complex and multifactorial. Hereditary syndromes, such as familial adenomatous polyposis and hereditary nonpolyposis CRC, account for <10% of all cases [2]. The majority of cases are thought to be caused by multiple factors, which include dietary and lifestyle habits and/or mild genetic predisposition [3]. Colorectal adenoma (CRA) is a recognized precursor of CRC based on epidemiologic, histological, and genetic studies demonstrating shared genetic alterations [4], [5].

Low dietary intake or plasma level of folate has been found to increase the risk of colorectal cancer (CRC) in several case–control and cohort studies [6]–[8]. Folate is essential for the synthesis of S-adenosyl-methionine, which is the methyl donor required for various methylation reactions in cells [9]. Methylation of CpG sites inhibits DNA transcription and regulates gene expression and imbalanced DNA methylation is observed consistently in colonic neoplasia [10], [11]. Accumulating evidence indicates that DNA methylation plays an important role in colorectal carcinogenesis [12].

Methionine synthase (MTR), on chromosome 1q43, encodes one of several key enzymes involved in the folate-mediated one-carbon metabolism. It catalyzes the methylation of homocysteine to methionine with simultaneous conversion of 5-methyl-tetrahydrofolate (5-methyl-THF) to tetrahydrofolate (THF). MTR is essential for the provision of S-adenosyl-methionine, the universal donor of methyl groups, as well as the provision of THF for use in nucleotide synthesis [13]. A common MTR variant consists of an A-to-G transition at base-pair 2756 and leads to a change from aspartic acid to glycine at codon 919 (D919G) [14]. Although the direct functional impact of this polymorphism has not been established, there is some evidence that this may be an activating polymorphism; in some studies, individuals with GG genotype have higher serum folate concentrations [15] and lower homocysteine concentrations [16], [17].

An association between MTR A2756G polymorphism and genetic susceptibility to CRC and CRA has been widely documented but with inconsistent results. A single study may be too underpowered to detect a possible small effect of the polymorphism on CRC/CRA, especially when the sample size is relatively small. Besides, different types of study populations and study design may also contribute to the disparate findings. To help clarify the inconsistent findings, we conducted a comprehensive meta-analysis to quantify the overall risk of MTR A2756G polymorphism on developing CRC/CRA.

## Materials and Methods

### Literature Search Strategy

Eligible literatures published before the end of September 2012 were identified by a search of PUBMED, EMBASE, Web of science and CNKI (China National Knowledge Infrastructure) databases. Search term combinations were keywords relating to the methionine synthase (e.g., “methionine synthase”, “MTR”, “one-carbon metabolism”) in combination with words related to CRC/CRA (e.g., “colorectal cancer”, “colorectal tumor”, “colorectal carcinoma”, “rectal cancer”, “colon cancer”, and “colorectal adenoma”) and “polymorphism” or “ variation”. All the searched studies were retrieved, and their references including relevant reviews were also hand-searched as well for other relevant studies. If more than one article were published using the same case series, only the study with largest sample size was selected.

### Selection Criteria and Data Extraction

Studies included in the meta-analysis had to meet all the following criteria: (1) original papers containing independent data, (2) identification of CRC/CRA was confirmed pathologically or histologically, (3) sufficient data to calculate the odds ratio (OR) with its 95% confidence interval (CI) and P value, and (4) case–control or cohort studies and (5) genotype distribution of the control population must be in Hardy–Weinberg equilibrium.

Two investigators extracted data independently. When it came to conflicting evaluations, an agreement was reached after a discussion among all authors. Data were collected on the first author’s surname, publication year, ethnicity of studied population (were categorized as ‘’ group), sample size, tumor site (rectal cancer vs. colon cancer), mean age of cases and controls, gender distribution in cases and controls, genotyping method, cigarette smoking status, alcohol consumption, confirmation of diagnosis, Hardy–Weinberg equilibrium (HWE) status, and genotype frequency in cases and controls. Where essential information was not presented in articles, every effort was made to contact the authors.

### Statistical Methods

For the MTR gene, we estimated the risks of G allele of A2756G on CRC/CAR, compared with the A allele. Then, we estimated the risks of the heterozygous and homozygote genotypes on CRC/CAR, compared with the wild-type AA homozygote. The strength of the association between the MTR gene and CRC/CRA risk was measured by ORs with 95% CIs. Cochran’s chi-square Q test was used to calculate heterogeneity across individual studies. Random-effects and fixed-effect summary measures were calculated as inverse-variance-weighted average of the log OR [18]. The results of random-effects summary were reported in the text which takes into account the variation between studies. The significance of the overall OR was determined by the Z-test. Study size (≥500, and <500 cases), source of controls (population versus hospital based), ethnicity (East Asian, Caucasian and others), sex, tumor sites (colon cancer versus rectum cancer) and types of end points (CRC versus CRA) were prespecified as characteristics for assessment of heterogeneity. Ethnic group was defined as East Asian (e.g., Chinese, Japanese, Korean), Caucasian (i.e., people of European origin), and Others ethnic populations (mixed or unknown populations). Subsequently, meta-regression was performed to further investigate potential sources of heterogeneity-ethnicity, sample size, age, and gender, specifying the method for estimating the between-study variance as restricted maximum likelihood. We assessed publication bias by using an ancillary procedure attributed to Egger et al. [19], which uses a linear regression approach to measure funnel plot asymmetry. To measure sensitivity analysis, each study was removed in turn from the total, and the remainder reanalyzed. This procedure was used to ensure that no individual study was entirely responsible for the combined results. All statistical analyses were carried out with the Stata software version 10.0 (Stata Corporation, College Station, TX). All P values are two-sided at the P = 0.05 level.

## Results

### Characteristics of Studies

Figure S1 shows the study selection process. A total of 27 studies were retrieved based on the search criteria for CRC/CRA susceptibility related to the MTR A2756G polymorphism [20]–[46]. The main study characteristics were summarized in Table 1. There are 22 studies with 13465 patients and 20430 controls concerning CRC and 6 studies with 4844 patients and 11743 controls concerning CRA. The genotype distributions in the controls for all studies were consistent with HWE. Characteristics of studies included in the current meta-analysis are presented in Table 1.

**Table 1:** Characteristics of the studies included in the meta-analysis.

Study	Year	Ethnicity	Cases	Controls source	Genotypingmethod	No. ofcases/controls	Mean age ofcases/controls	Sex distribution in cases/controls (male %)
Ma [20]	1999	American	CRC	Population	RFLP	356/476	NA/NA	100/100
Matsuo [21]	2002	Japanese	CRC	Hospital	RFLP	142/241	NA/NA	58.9/49.0
Le Marchand [22]	2002	American	CRC	Population	RFLP	539/652	66.0/67.0	60.8/57.9
Ulvik [23]	2004	Norwegian	CRC	Population	Taqman	2168/2192	NA/NA	63.5/63.5
Ulrich [24]	2005	American	CRC	Population	Taqman	1600/1962	64.9/65.0	56.0/53.0
Matsuo [25]	2005	Japanese	CRC	Hospital	RFLP	257/771	58.8/59.0	63.0/63.0
Chen [26]	2006	Chinese	CRC	Population	RFLP	199/413	62.5/61.9	50.8/51.7
Koushik [27]	2006	American	CRC	Population	Taqman	363/804	68.2/68.0	47.6/42.0
Curtin [28]	2007	American	CRC	Population	Taqman	916/1974	NA/NA	NA/NA
Steck [29]	2008	American	CRC	Population	Taqman	546/855	63.8/65.9	NA/NA
Zhang [30]	2008	Chinese	CRC	Hospital	RFLP	298/300	57.7/57.6	56.3/56.7
Guerreiro [31]	2008	Portuguese	CRC	Population	Taqman	196/200	64.2/62.2	53.1/53.0
Theodoratou [32]	2008	Scottish	CRC	Population	Array	999/1010	62.3/62.7	57.3/56.9
Zhang [33]	2009	Chinese	CRC	Hospital	RFLP	476/835	54.3/52.0	57.1/55.1
de Vogel [34]	2009	Dutch	CRC	Population	SNaPShot	696/1805	NA/NA	55.0/50.2
Levine [35]	2010	American	CRC	Population	iPLEX	1806/2879	53.5/54.0	51.3/44.4
Eussen [36]	2010	European	CRC	Population	Mass spectrometry	1329/2364	58.9/58.7	51.0/53.0
Guimarães [37]	2011	Brazilian	CRC	Population	RFLP	113/188	59.0/54.0	53.1/64.4
Jokić [38]	2011	Croatian	CRC	Population	Taqman	300/300	62.2/61.4	54.0/50.6
Kim [39]	2011	Korean	CRC	Hospital	RFLP	67/53	61.8/58.7	52.2/43.4
Martinelli [40]	2012	Italian	CRC	Population	RFLP	71/80	69.0/58.0	59.2/53.8
Pufulete [41]	2003	British	CRC, CRA	Hospital	RFLP	63/76	68.9/58.0	46.0/45.0
Goode [42]	2004	American	CRA	Population	Taqman	513/609	NA/NA	63.0/38.8
Hazra [43]	2007	American	CRA	Population	Taqman	529/532	NA/NA	0/0
Yamaji [44]	2009	Japanese	CRA	Population	Taqman	723/607	61.0/60.0	67.5/65.4
de Vogel [45]	2011	Norwegian	CRA	Population	Mass spectrometry	1713/8355	56.9/56.1	60.6/47.0
Han [46]	2012	American	CRA	Population	Taqman	1331/1501	NA/NA	NA/NA

### Meta-analysis Results

**Figure 1:**
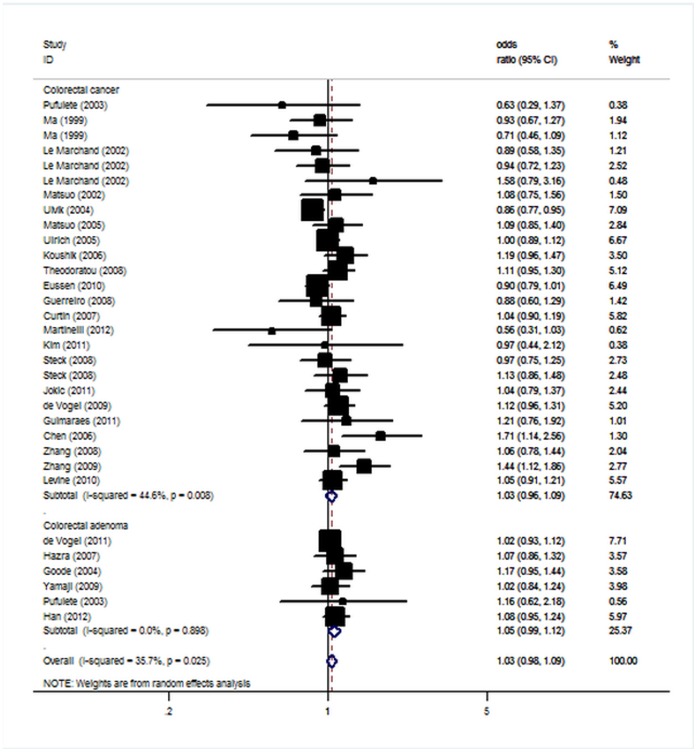
Forest plot from the meta-analysis of MTR A2756G polymorphism and CRC/CRA risk.

As shown in Figure 1 and Table 2, no significant associations between the A2756G polymorphism of MTR and CRC or CRA susceptibility were found.

**Table 2:** Main results of pooled odds ratios (ORs) with confidence interval (CI) in the meta-analysis.

Sub-group analysis	No. of cases/controls	G Allele			Heterozygous			Homozygous		
		OR (95%CI)	P(Z)	P(Q)	OR (95%CI)	P(Z)	P(Q)	OR (95%CI)	P(Z)	P(Q)
Colorectal cancer	13465/20430	1.03 (0.96–1.09)	0.42	0.008	1.04 (0.96–1.12)	0.36	0.03	0.99 (0.85–1.16)	0.91	0.19
**Ethnicity**										
East Asian	1754/3007	1.17 (1.00–1.36)	0.06	0.14	1.19 (0.96–1.48)	0.11	0.05	1.22 (0.84–1.78)	0.30	0.78
Caucasian	11396/17014	0.99 (0.92–1.05)	0.66	0.05	0.99 (0.92–1.07)	0.82	0.20	0.93 (0.77–1.13)	0.46	0.09
Others	315/409	1.18 (0.92–1.52)	0.20	0.37	1.13 (0.82–1.55)	0.46	0.40	1.57 (0.78–3.19)	0.21	0.69
Sample size										
Large studies	9514/14186	0.99 (0.92–1.08)	0.90	0.02	0.99 (0.90–1.10)	0.90	0.03	0.95 (0.73–1.25)	0.73	0.008
Small studies	3951/6244	1.06 (0.96–1.16)	0.28	0.08	1.08 (0.97–1.20)	0.18	0.16	1.09 (0.86–1.37)	0.48	0.79
**Controls source**										
Population	12197/18154	1.01 (0.94–1.08)	0.81	0.01	1.02 (0.94–1.10)	0.71	0.05	0.96 (0.81–1.14)	0.67	0.16
Hospital	1268/2276	1.14 (0.97–1.33)	0.12	0.29	1.15 (0.94–1.40)	0.17	0.25	1.27 (0.82–1.97)	0.28	0.50
Tumor sites										
Colon cancer	1988/2779	1.06 (0.88–1.28)	0.56	0.10	1.09 (0.88–1.35)	0.41	0.15	0.78 (0.56–1.09)	0.15	0.62
Rectum cancer	1331/2162	1.24 (0.88–1.74)	0.21	0.002	1.32 (0.93–1.89)	0.12	0.008	0.77 (0.48–1.23)	0.28	0.58
**Sex**										
Male	3004/3810	0.92 (0.82–1.02)	0.12	0.23	0.95 (0.81–1.11)	0.53	0.13	0.75 (0.51–1.10)	0.14	0.12
Female	1796/2623	1.02 (0.86–1.22)	0.81	0.07	1.01 (0.89–1.16)	0.86	0.59	1.03 (0.61–1.74)	0.92	0.05
Colorectal adenoma	4844/11743	1.05 (0.99–1.12)	0.11	0.89	1.03 (0.94–1.12)	0.57	0.80	1.16 (0.94–1.43)	0.18	0.52
Caucasian only	4121/11073	1.06 (0.99–1.13)	0.11	0.83	1.02 (0.92–1.12)	0.76	0.73	1.21 (0.97–1.52)	0.09	0.60
Large studies only	4809/11667	1.05 (0.99–1.12)	0.12	0.82	1.02 (0.93–1.12)	0.64	0.85	1.17 (0.93–1.46)	0.18	0.37
Total	18309/32173	1.03 (0.98–1.09)	0.19	0.03	1.03 (0.97–1.10)	0.28	0.08	1.03 (0.90–1.17)	0.68	0.20

In the overall analysis, the 2756G was not significantly associated with elevated CRC risk. Using random effect model, the per-allele overall OR of the G variant for CRC was 1.03 (95% CI: 0.96–1.09, P = 0.42), with corresponding results for heterozygous and homozygous of 1.04 (95% CI: 0.96–1.12, P = 0.36) and 0.99 (95% CI: 0.85–1.16, P = 0.91), respectively. This analysis is based on pooling of data from a number of different ethnic populations. When stratifying for ethnicity, an OR of 0.99 (95% CI: 0.92–1.05, P = 0.66) and 1.17 (95% CI: 1.00–1.36, P = 0.06) resulted for G allele, among Caucasians and East Asians, respectively. By considering control source subgroups, the OR was 1.01 (95% CI: 0.94–1.08, P = 0.81) in population-based controls compared to 1.14 (95% CI: 0.97–1.33, P = 0.12) in hospital controls. In the stratified analysis by sample size, no significant associations were found in large studies or small studies. In the subgroup analyses by tumor site, no significant associations were found for colon cancer and rectum cancer in all genetic modes. Subsidiary analyses of sex yielded a per-allele OR for male patients of 0.92 (95% CI: 0.82–1.02, P = 0.12) and for female patients of 1.02 (95% CI: 0.86–1.22, P = 0.81). Similar results were also detected for heterozygous and homozygous. In a meta-regression analysis, neither the ethnicity, sample size, nor age, sex correlated with the magnitude of the genetic effect (P>0.05 for all).

Data on genotypes of the MTR A2756G polymorphism among cases and controls stratified by smoking and alcohol consumption were available in four studies. Among heavy smokers (≥40 package/year) in all four studies, G allele carriers had a significantly increased CRC risk compared to the wild AA genotype with an OR of 2.06 (95% CI: 1.32–3.20; P = 0.001, P _heterogeneity_ = 0.29). Using dominant genetic model, heavy alcohol drinkers (≥50 g ethanol/d on ≥5 day/week) with the G allele of A2756G variant had a significantly increased CRC risk with an OR of 2.00 (95% CI: 1.28–3.09; P = 0.002, P _heterogeneity_ = 0.38).

Overall, there was no evidence for the association between MTR A2756G and CRA risk (G allele: OR = 1.05, 95% CI: 0.99–1.12; heterozygous: OR = 1.03, 95% CI: 0.94–1.12; homozygous: OR = 1.16, 95% CI: 0.94–1.43). No significant heterogeneity was found for included studies (P>0.05). Analysis restricted to the 4 studies with at least 500 cases, yielded an OR of 1.05 (95% CI: 0.99–1.12) for G allele.

### Sensitivity Analyses and Publication Bias

A single study involved in the meta-analysis was deleted each time to reflect the inﬂuence of the individual data-set to the pooled OR, and the corresponding pooled OR was not materially altered, suggesting that the results of this meta-analysis are stable (data not shown). As shown in Figure S2, the shape of the funnel plot seemed symmetrical, suggesting no publication bias among the studies included. Egger’s test also indicated no evidence of publication bias in relation to allele or genotype comparison (P>0.05, for all).

## Discussion

Genes in the one-carbon metabolic pathway may modulate risk of CRC and CRA by influencing methyl group availability for DNA methylation reactions or nucleotide synthesis [11], [28], [34], [47], [48]. It is currently believed that perhaps up to 30% of colorectal cancer cancers are characterized by this CpG island methylator phenotype (CIMP), in which numerous CpG islands are methylated and tumor suppressor genes such as the cell-cycle regulator, p16, are inactivated [49], [50]. A recent study showed that 60% of colorectal adenomas are abnormally methylated on at least one locus. Furthermore, CpG island methylation phenotype has been demonstrated in large colorectal adenomas and in adenomas with tubular or villous histology [51], [52]. This is the most comprehensive meta-analysis examined the A2756G polymorphism of MTR and the relationship to susceptibility for CRC and CRA. Its strength was based on the accumulation of published data giving greater information to detect significant differences. In total, the meta-analysis involved 26 studies which provided 18309 cases and 32034 controls.

In this large-scale meta-analysis, the combined evidence suggested that MTR A2756G polymorphism did not contribute to the development of CRC or CRA. For the subgroup analysis based on ethnicity, sample size, source of controls, tumor site, and sex we were unable to observe any effect modification, which is in line with the pooled analysis. Data from the studies did not exhibit statistically significant heterogeneity in the majority of contrasts. There are some possible reasons for the inconsistent results in early reports. Firstly, ethnic differences may attribute to these different results, since the distributions of the A2756G polymorphism were different between various ethnic populations. In fact, the distribution of the less common G allele varies extensively between different races, with a prevalence of ∼14% among East Asians, ∼20% among Caucasians. On the other hand, study design or small sample size or some environmental factors may affect the results. Most of these studies did not consider most of the important environmental factors. It is possible that variation at this locus has modest effects on CRC/CRA, but environmental factors may predominate in the progress of CRC/CRA, and mask the effects of this variation. Specific environmental factors like lifestyle and cigarette smoking have already been well studied in recent decades [53], [54]. The unconsidered factors mixed together may cover the role of MTR polymorphism. Thus, even if the variation has a causal effect on CRC, it may take a long time to be observed. CRC/CRA is a complex disease, and both environmental and genetic factors are involved in the development of the disease. Thus, the effect of single genetic factor on the risk of CRC/CRA may be more pronounced in the presence of other common genetic or environmental risk factors such as smoking, red meat consumption, and alcohol abuse.

Alcohol consumption has a hazardous effect on folate metabolism, which is closely related to the DNA methylation pathway. Unfortunately, almost all the studies failed to explore the interaction between MTR genotype and alcohol drinking habits. This was probably due to the low statistical power of the individual studies to detect interactions. Our results show a significant association among heavy drinkers subgroup between MTR A2756G polymorphism and CRC risk. Alcohol drinking inhibits folate absorption from the intestine, interrupts folate release from the liver, and promotes urinary excretion of folate from the body [55], all of which result in reduced levels of serum folate [56], of which 5-methyl-tetrahydrofolate is the predominant component. The provision of 5-methyltetrahydrofolate is crucial for methionine synthase to catalyze the remethylation of homocysteine to methionine. In addition, alcohol intake can directly interfere with the enzymatic activity of methionine synthase [57]. Further, the influence of alcohol intake may vary among individuals with different genetic susceptibility. Our observation of an interaction between MTR A2756G and alcohol consumption suggests that the DNA methylation pathway is implicated in the development of colorectal cancer. Tobacco smoking is an established risk factor for many cancers. By pooling the collected data on smoking status and MTR genotypes, a statistically significant 2.00 fold increased risk for CRC appeared for individuals carrying the risk G allele in comparison with individuals with the wild AA genotype. This result suggests that in the presence of both of the two risk factors, an important number of CRC cases would occur.

Some limitations should be considered when interpreting our results, in addition to those inherited from the meta-analysis. Firstly, the subgroup meta-analyses considering interactions between MTR genotype and cigarette smoking, as well as alcohol drinking, were performed on the basis of a fraction of all the possible data to be pooled, so selection bias may have occurred and our results may be over inﬂated. Secondly, our results were based on unadjusted estimates, whereas a more precise analysis could be conducted if all individual raw data were available, which would allow for the adjustment by other co-variables including age, sex, drinking status, cigarette consumption, and other lifestyle factors. Ideally we would like to pool individual-level data. However this is not possible for the present study. Further large and well-designed studies need to be performed to further confirm our results. Thirdly, because only published studies were retrieved in the meta-analysis, publication bias might be possible, even though the statistical test did not show it.

In conclusion, our meta-analysis suggests that there is no association between MTR A2756G polymorphism and CRC/CRA. However, the A2756G polymorphism of MTR may modulate the tobacco-related as well as alcohol-related pathogenesis of CRC. For future association studies, strict selection of patients and larger sample size of different ethnic populations are required. Moreover, gene–gene and gene–environment interactions should also be considered.

## Supporting Information

Figure S1 **Study selection process.**
(TIF)

Figure S2 **Funnel plot of association between MTR A2756G polymorphism and CRC/CRA overall individuals.**
(TIF)

Checklist S1 **PRISMA Checklist.**
(DOC)
