# Supplementary figures and images for: Methionine Synthase A2756G Polymorphism and Risk of Colorectal Adenoma and Cancer: Evidence Based on 27 Studies
Source: PLoS One. 2013 Apr 9;8(4):e60508. doi: 10.1371/journal.pone.0060508 (PMC3621882; doi:10.1371/journal.pone.0060508)

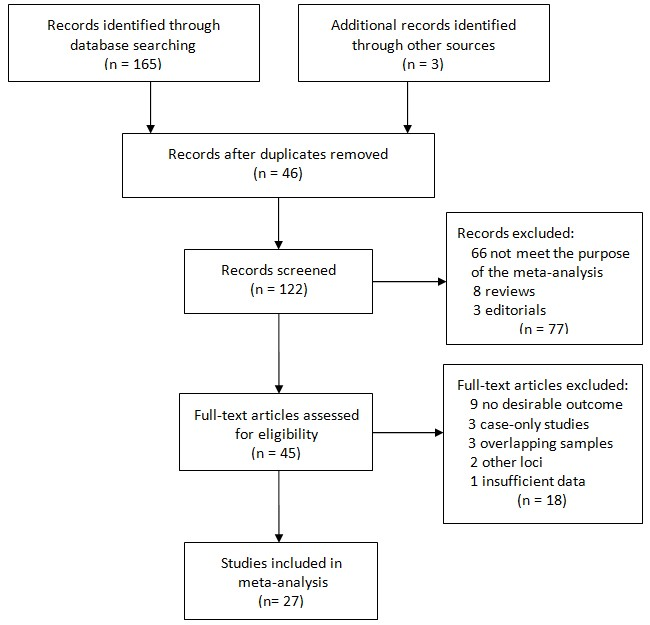

Supplement: Figure S1 — Study selection process. (TIF) [file pone.0060508.s001.tif]

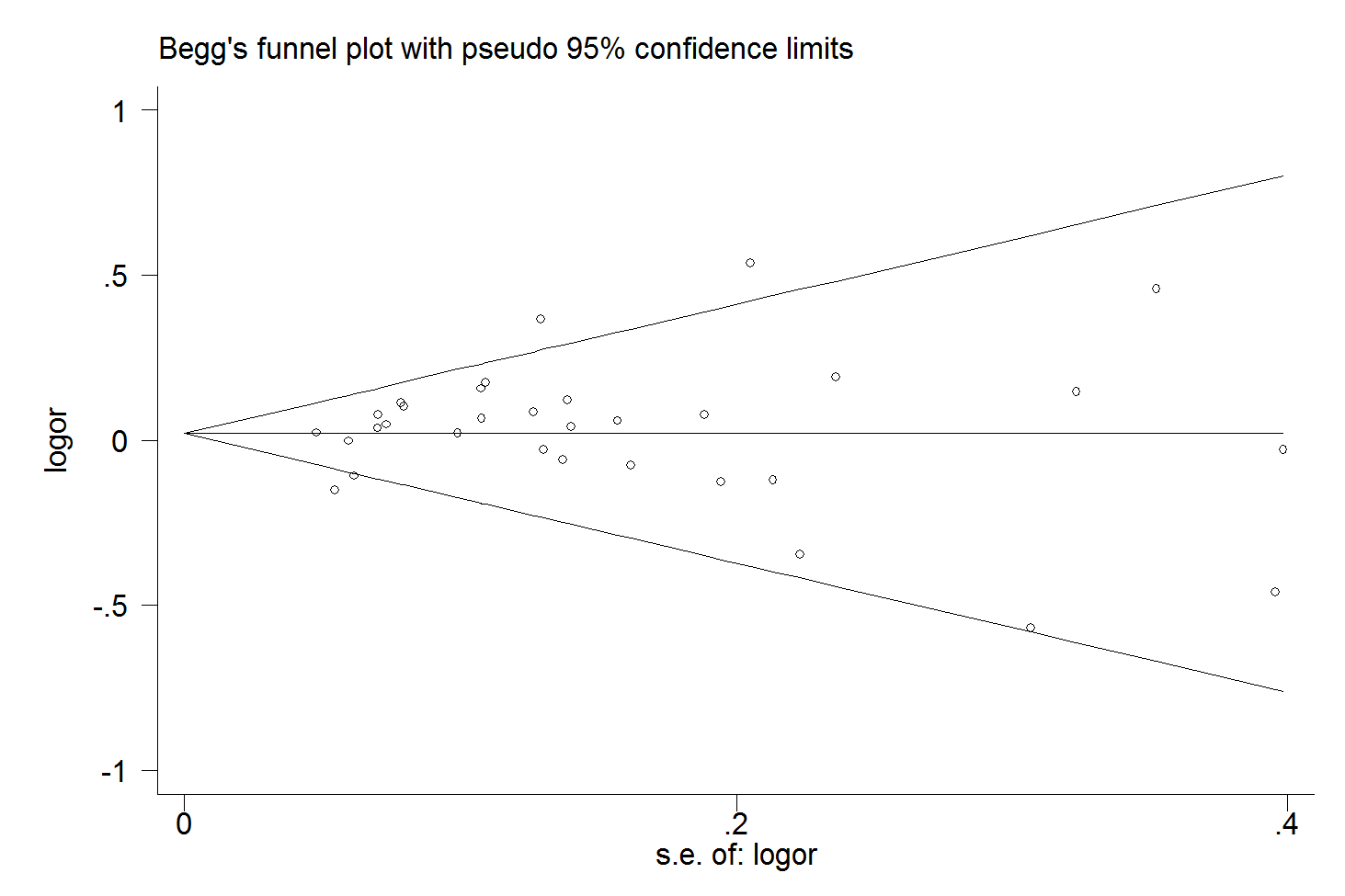

Supplement: Figure S2 — Funnel plot of association between MTR A2756G polymorphism and CRC/CRA overall individuals. (TIF) [file pone.0060508.s002.tif]
